# The significance of eosinophils in predicting the severity of acute ischemic stroke

**DOI:** 10.18632/oncotarget.22199

**Published:** 2017-10-31

**Authors:** Jun Wang, Li Ma, Tao Lin, Shi-Jing Li, Lei-Lei Chen, De-Zhao Wang

**Affiliations:** ^1^ Department of Cardiology, Beijing Mentougou District Hospital, Beijing 102300, China; ^2^ Department of Cardiology, Beijing Tiantan Hospital, Capital Medical University, Beijing 100050, China

**Keywords:** eosinophil, acute ischemic stroke, national institute of health stroke scale

## Abstract

**Background:**

Previous studies have shown that tumor-associated tissue eosinophilia have a role in various types of solid tumors. However, the relationship between eosinophil and acute ischemic stroke (AIS) is unclear. We aimed to investigate the diagnostic significance of eosinophil in AIS patients.

**Methods:**

This study included 300 AIS patients without hypereosinophilic syndrome (HES). The hematologic indices were collected from each patient, including white blood count, eosinophil count, eosinophil percentage, neutrophil count, red blood count, and platelet. The severity of AIS was estimated by national institute of health stroke scale (NIHSS). Logistic regression analyses were performed to confirm the biomarkers for NIHSS and in-hospital non-death among the cases. Moreover, receiver-operating characteristics (ROC) analyses were used to investigate the clinical performances of eosinophils and NIHSS in prediction of non-death.

**Results:**

The admission NIHSS (*P*<0.001) and BMI (*P*<0.001) were predictors to the non-death of the patients. There was a significant correlation between eosinophil counts or eosinophil percentage and NIHSS score (r= -0.451, *P* < 0.001; r= -0.617, *P<*0.001, Spearson Correlation). ROC analysis showed that eosinophil counts and eosinophil percentage could predict non-death of the patients in-hospital, with the areas under the curves (AUC) of 0.791 and 0.867, respectively.

**Conclusions:**

Our study revealed a relationship between eosinophil and NIHSS score in the patients with AIS. Eosinophils might have certain value for predicting the severity of AIS.

## INTRODUCTION

Stroke is one of the leading causes of death and adult disability in the United States [1]. Atherosclerosis is the main cause of stroke and a lipoproten-driven disease that leads to plaque formation at specific site of the arterial tree through intimal inflammation, necrosis, fibrosis, and calcification. The magnitude of the thrombotic response on ruptured or eroded plaques is extremely variable, and only occasionally does a major cerebral artery thrombus evolve. Inflammation plays an important role in the process of plaque rupture and the formation of thrombosis [2]. Besides, circulating monocytes are characterized by refractory state or endotoxin tolerance which could be employed as predictors of ischemic stroke [3]. For example, Pagram H et al. showed that peripheral neutrophils have potential as a biomarker for immunomodulatory therapy in patients with a severe stroke [4].

Eosinophils are specialized inflammatory cells that mediate pathologic tissue damage and protect the host against metazoan parasites. The dysregulation of eosinophils may cause inflammatory disorders, thus leading to human diseases, especially the hypereosinophilic syndrome (HES) [5, 6]. Brain infarction is the frequent patterns of neurological involvement in HES. Brain infarcts are attributed to thromboembolism from endomyocardial fibrosis or vascular endothelial toxicity of eosinophilic cells in patients with HES [7, 8]. Now most of the studies investigate the association of eosinophils and brain infarction in acute ischemic stroke (AIS) with HES, few studies are focused on AIS without HES. As far as we know, the clinical role of eosinophils in AIS without HES is still not clear.

Therefore, in the present study, we expected to explore the clinical significance of eosinophil in acute ischemic stroke (AIS) patients without HES.

## RESULTS

### Characteristics at baseline

Baseline characteristics of the subjects were shown in Table 1. A total of 300 eligible patients were recruited in our study, including 187 males (62.3%) and 113 females (37.7%). The average age was 63.7 ± 9.5 years old, and their, body mass index (BMI) was 26.7 ± 3.2 kg/m^2^, The other parameters of the cases were also summarized in the table, including white blood counts, lymphocytes, eosinophil counts, eosinophil percentage, red blood counts, admission NIHSS, NIHSS discharge, etc.

**Table 1:** Baseline clinical characteristics

Characteristics	Over all
	n=300
Age (years)	63.7±9.5
Male (n, %)	187 (62.3)
BMI (kg/m^2^)	26.7±3.2
Hypertention (n, %)	198 (66.0)
Hyperlipemia (n, %)	57 (19.0)
Diabetes mellitus (n, %)	108 (36.0)
Smoke (n, %)	118 (39.3)
WBC (10^9^/L)	7.21±2.26
Lymphocytes (10^9^/L)	1.78±0.50
Monocytes (10^9^/L)	5.82±1.67
Neutrophils (10^9^/L)	4.87±2.06
Eosinophils (10^9^/L)	0.14±0.12
Basophils	0.03±0.02
Eosinophil percentage (%)	1.45 (0.70-2.30)
RBC (10^12^/L)	4.72±0.58
Platelet (10^9^/L)	228.5±83.4
LDL-c (mmol/L)	2.69±0.67
Creatinine (umol/L)	74.2±22.6
On admission NIHSS (score)	6.34±4.30
Discharge NIHSS (score)	3.71±3.88
Infarction related artery (n, %)	
ICA	124 (41.3)
VBA	132 (44.0)
ICA and VBA	44 (14.7)
Death (n, %)	14 (4.7)

The value are mean ±SD.

Abbreviations: BMI, body mass index; WBC, white blood count; RBC, red blood count; LDL-c, low-density lipoprotein cholesterol; NIHSS, national institute of health stroke scale; ICA, internal carotid artery; VBA, vertebral basilar artery.

### Multi-factorial analysis of patients in-hospital death

The backward stepwise logistic regression analysis was performed to analyze the predicted factors for in-hospital death among the study subjects. The results showed that admission NIHSS (OR, 2.188; 95% CI, 1.504-3.182; *P*<0.001) and BMI (OR, 0.139; 95% CI, 0.052-0.373; *P*<0.001) were significantly correlated with in-hospital death among the cases, which could be employed as biomarkers (Table 2).

**Table 2:** Multivariate logistic regression analysis of variable related to death

Category	B	S.E.	Wald	OR (95%CI)	*P* value
Admission NIHSS score	0.783	0.191	16.779	2.188(1.504-3.182)	<.001
BMI	-1.974	0.504	15.331	0.139(0.052-0.373)	<.001

Method=backward stepwise (wald).

Abbreviations: NIHSS, national institute of health stroke scale; OR, odds ratio; CI, confidence interval.

Model adjusted for sex, age, body mass index, on admission NIHSS, hypertension, hyperlipemia, diabetes mellitus, smoke, eosinophils, low-density lipoprotein cholesterol, and discharge NIHSS.

### Correlation between NIHSS and baseline characteristics

NIHSS showed correlation with age (*r* = 0.148), gender (*r* = -14.516), hypertension (*r* = -14.847), hyperlipemia (*r* = -14.925), diabetes mellitus (*r* = -14.754), smoke (*r* = -14.673), white blood counts (*r*=0.191), lymphocytes (*r* = -0.214), eosinophils (*r* = -0.451), eosinophil percentage (*r* = -0.617), and low-density lipoprotein cholesterol (*r* = -0.261) (Table 3).

**Table 3:** Correlation between NIHSS and clinical characteristics of the patients

Abbreviations	NIHSS	
	r value(Spearson or Wilcoxon)	*P* value
Age	0.148	0.010
Gender	-14.516	<.001
BMI	0.039	0.499
Hypertension	-14.847	<.001
Hyperlipemia	-14.925	<.001
Diabetes Mellitus	-14.754	<.001
Smoke	-14.643	<.001
WBC	0.191	0.001
Lymphocytes	-0.214	<.001
Eosinophils	-0.451	<.001
Eosinophil percentage	-0.617	<.001
RBC	0.029	0.612
Platelet	0.045	0.440
LDL-c	-0.261	<.001

Abbreviations: BMI, body mass index; WBC, white blood count; RBC, red blood count; LDL-c, low-density lipoprotein cholesterol; NIHSS, national institute of health stroke scale.

### Indicators for NIHSS among the study subjects

In order to identify the indicators for NIHSS in the AIS patients without HES, the multivariate linear regression analysis was performed. Analysis results demonstrated that eosinophils (95% CI, -31.130 - -14.528; *P*<0.001), lymphocytes (95% CI, -3.586 - -2.066; *P*<0.001), white blood counts (95% CI, 1.412-1.881; *P*<0.001), body mass index (95% CI, -0.978- -0.647; *P*<0.001), smoke (95% CI, -3.379- -1.946; *P*<0.001), diabetes mellitus (95% CI, 0.497-2.019; *P*=0.001), and eosinophil percentage (95% CI, 0.014-0.789; *P*=0.042) were predictors for NIHSS (Table 4).

**Table 4:** Predictors of NIHSS in multivariate linear regression analysis (Method: Stepwise).

Model	Unstandardized Coefficients		Standardized Coefficients	t	Sig.	95%CI
	B	Std.Error	Beta			
(constant)	23.999	1.856		12.930	<.001	20.346-27.651
Eosinophils	-22.829	4.218	-0.645	-5.413	<.001	-31.130--14.528
Lymphocytes	-2.826	0.386	-0.325	-7.318	<.001	-3.586--2.066
WBC	1.647	0.119	0.864	13.829	<.001	1.412-1.881
BMI	-0.812	0.084	-0.601	-9.661	<.001	-0.978--0.647
Smoke	-2.662	0.346	-0.303	-7.310	<.001	-3.379--1.946
Diabetes Mellitus	1.258	0.387	0.141	3.252	0.001	0.497-2.019
Eosinophil percentage	0.402	0.197	0.264	2.042	0.042	0.014-0.789

Abbreviations: CI, confidence interval; NIHSS, national institute of health stroke scale; WBC, white blood count; BMI, body mass index.

Model adjusted for age, gender, body mass index, smoke, hypertension, hyperlipemia, diabetes mellitus, white blood count, lymphocytes, eosinophils, eosinophil percentage, and low-density lipoprotein cholesterol.

### The predictive values of eosinophils and eosinophil percentage

**Figure 1:**
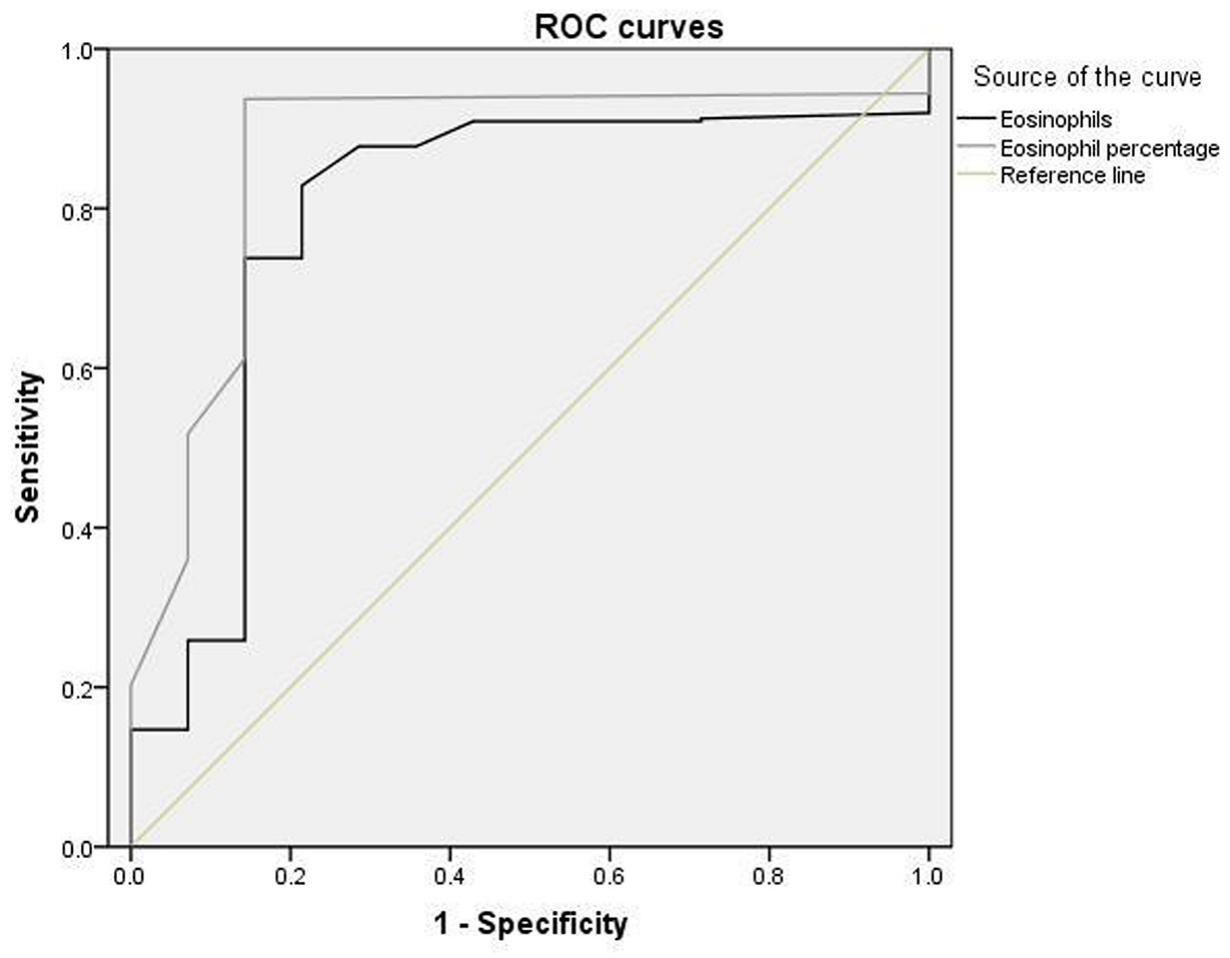
The receiver-operating characteristic(ROC) analysis for Eosinophils and Eosinophil percentage in predicting non-death

In the current study, ROC curve was plotted to estimate the clinical performance of eosinophils and eosinophil percentage in predicting in-hospital non-death among the study subjects. Eosinophils counts of 0.045×10^9^/L could independently predict the non-death in AIS patients without HES, with the sensitivity of 82.9% and the specificity of 78.5% (AUC=0.791; 95%CI: 0.659-0.923). Furthermore, eosinophil percentage could also be employed as a biomarker for non-death among the study population (AUC=0.867; 95%CI: 0.764-0.969). The cut-off value was of 0.30%, with the sensitivity of 93.7% and the specificity of 85.7% (Table 5 and Figure 1).

**Table 5:** Area under curve(AUC), Sensitivity, and Specificity

Variable	Area under curve		Sensitivity	Specificity	cut-off value
	AUC (95%CI)	*P* value			
Eosinophil counts	0.791(0.659-0.923)	<.001	0.829	0.785	0.045×10^9^/L
Eosinophil percentage	0.867(0.764-0.969)	<.001	0.937	0.857	0.30

Abbreviations: AUC, area under curve; CI, confidence interval.

## DISCUSSION

In this study, we found the association between decreased eosinophils or eosinophil percentage and increased risk of acute cerabral infarction in AIS patients without HES. NIHSS is an important scale for predicting the severity of acute cerabral infarction [9–11]. NIHSS score was negatively related to eosinophils or eosinophil percentage in our study. Eosinophils or eosinophil percentage might be employed as biomarkers to predict the severity of cerebral infarction.

HES and Churg-Strauss syndrome (CSS) are the diseases characterized by extreme eosinophilia. HES is a systemic hematologic order characterized by multiorgan system involvement such as endocarditis [5, 12]. Eosinophilic endocarditis is characterized by massive infiltration into the endocardium and myocardium of activated eosinophils and mural thrombosis leading to systemic embolism such as hepatic cerebral sinus thrombosis. Hypothiocyanous acid generated by adherent and infiltrating eosinophils may provoke the development of a prothrombotic and proinflammatory endothelial/endocardial phenotype that promotes the pronouned thrombotic diathesis [5]. CSS also known as extreme eosinophilia and multiple cerebral vascular lesions is a form of vasiculitis characterized by inflammation of the smaller arteries, arterioles, and venules [13]. Vasculitis lesions in brain can lead to multiple cerebral infarction. Most previous studies have showed that the cytotoxic effect of proteins(including major basic protein (MBP), eosinophil peroxidase (EPOX), eosinophil cationic protein (ECP), and eosinophil-derived neurotoxin (EDN)) released by circulating eosinophils, thrombus formation, and endothelial damage are causes of brain infarction. Thromboembolic infarction and direct eosinophilic toxicity may be caused by hypereosinophilia in cerebral arteries [14]. The main mechanism of eosinophilic toxicity to cerebral arteriolar endothelial cells was though the production of tumor necrosis factor-α (TNF-α) mediated by eosinophil cationic granular protein [15]. Is there a relationship between eosinophils and thrombosis in patients without HES or CSS?

Actually, there are a few studies focusing on eosinophils and coronary artery thrombosis. The study of Sakai *et al.* showed that eosinophil infiltration was predominantly observed in the area between white thrombus and red thrombus in 106 males that they performed histologic analysis of tissue samples obtained by thrombus aspiration therapy from patients with acute coronary syndrome [16]. Another study suggested that eosinophils presented in all samples from coronary arterial thrombi, and eosinophil percentage was decreased in peripheral blood in patients with acute myocardial infarction [17]. Histopathologic findings suggested that eosinophils might play a pivotal role in the formation of thrombi. The mechanism by which eosinophils was involved in coronary thrombosis might be eosinophils promoting thrombi formation by activating platelet and the powerful procoagulant activity of purified EPOX released by circulating eosinophils [6]. Eosinophils is decreased in patients with acute coronary syndrome, however is that right in patients with acute cerebral infarction without HES or CSS?

At present, we can not direct obtain the thrombus samples of infarction related artery in patients with acute cerebral infarction. So we can not get the direct evidence of relationship on eosinophils and thrombosis by the analysis of the thrombus in patients with acute ischemic stroke. NIHSS is developed as an impairment scale that is administered reliably by a variety of clinicians to evaluate stroke severity before and after treatment, primarily to evaluate the effects of intervention [10]. Use of a standardized scale helps to ensure that the neurological examination is performed in a timely fashion. These scores not only help to quantify the degree of neurological deficit but also facilitate communication between healthcare professionals, identify the possible location of vessel occlusion, provide early prognosis, and help to identify patient eligibility for various interventions and the potential for complications [18–20]. Our study showed that NIHSS was a predictor of prognosis of patients with acute cerebral infarction. This point was similar to the results of previous study. Then we analyzed the relationship between the various variables and NIHSS. NIHSS showed positive correlation with male, hypertension, hyperlipemia, diabetes mellitus, and smoke, and showed negative correlation with eosinophils and eosinophil percentage. The linear relationship between eosinophils and NIHSS revealed that eosinophils might play a certain role in the occurrence, development and prognosis of acute cerebral infarction.

The possible mechanism underlying the relationship between eosinophils and thrombus in patients with cerebral infarction is as follows. Firstly, the cytotoxic effect of proteins (including MBP, EPOX, ECP, and EDN) released by circulating eosinophils, is involved in the formation of blood clots. The crystalline structure of eosinophil MBP indicates that the protein specifically binds heparin [21]. EPOX reduces the anticoagulant effects of tryptase and heparin and clotting times. ECP enhances factor XII-dependent reactions, shortens the coagulation time of normal plasma, inactivate the anticoagulant activity of heparin, and inhibits the anticoagulant activity of glycosylated form of thrombomodulin [6, 14, 22]. Secondly, circulating eosinophils decreased under various environment such as acute inflammation and AMI [17, 23]. Our study showed that circulating eosinophils was decreased in patients with AIS. A rise in ECP during inflammatory disease with eosinophils decreased may be due to the localization of eosinophils in inflammatory lesions producing degranulation or disintergration and be released of ECP that can diffuse into the circulation. Alternatively ECP may be released from circulating eosinophils as a response to mediators which are activated in the inflammatory reaction. In the latter case eosinophils can not be recognized in the circulation [23]. Thirdly, EPOX accumulate at sites of eosinophil inflammation. Three unusual substrates such as bromide(Br^-^), nitrite(NO_2_^-^), and thiocyanate(SCN^-^) compete for oxidation by EPOX in the presence of H_2_O_2_ to yield hypobromous acid(HOBr), nitrogen dioxide(NO_2_), or hypothiocyanous acid(HOSCN) [24–26]. Three oxidant products have strikingly different reactivities: HOBr and NO_2_ are strong reactive membran-lytic oxidants, whereas HOSCN is a weak, sulfhydryl(SH)-specific oxidant penatrating into cells and imposing an intracellular oxidant stress [27, 28]. HOSCN generated by adherent and infiltrating eosinophils may provoke the development of a prothrombotic and proinflammatory endothelial/endocardial phenotype that promotes the pronounced thrombotic diathesis [5]. Finally, tissue factor(TF) plays a role in the pathology of thrombosis. Phagocyte oxidants such as H_2_O_2_ and HOSCN stimulate TF expression in endothelial and monocytes, and are relevant to eosinophil-mediated tissue damage [5, 29, 30].

### Limitations

The study has some limitations that should be considered. First, the study is not a prospective research. Second, because we can not obtain the thrombus samples of infarction related artery in patients with acute cerebral infarction, we do not get the direct evidence of relationship on eosinophils and thrombosis by the analysis of the thrombus. Third, we don’t consider the carotid lesions, which could produce potential biased results. Further research needs to be done. Even so our findings also have some value.

## MATERIALS AND METHODS

### Study design

This study was a retrospective analysis of a double-site study. The study was approved by the ethical committee of Beijing Tiantan Hospital, and written informed consent was gained from the participants. The study sites included those with experts in the fields of emergency medical services, primary care, radiology, emergency medicine (ED) and neurology.

### Study participants

The consecutive patients with AIS in Beijing Tiantan Hospital from January 1, 2014 to December 31, 2015 were selected. The patients with artery atherosclerotic cerebral infarction were conformed according to the patients history, physical examination, neurological examination and stroke scale, brain coputed tomography or brain magnetic resonace imaging, and access to neurological expertise [9]. Patients were excluded from the study if they had infectious diseases, eosinophils respiratory diseases, eosinophils skin diseases, eosinophils digestive tract diseases, tumor, eosinophils related immunology and rheumatology diseases, eosinophils related allergic diseases, hematology diseases, acute myocardial infarction, heart failure, any medication that can potentially interfere with the measurement of eosinophil counts, cerebral hemorrhage, subarachnoid hemorrage, transient ischemic attack, veins and venous sinus thrombosis, and severe lung disease. All the subjects underwent the following examinations including past history, physical examination, complete blood count, serum lipid, and national institutes of health stroke scale (NIHSS).

### Laboratory tests

Blood samples were collected on admission to casualty before any drug administration. Serum lipid was measured using an automated chemical analyzer (Olympus AU-600; Olympus, Tokyo, Japan), with reagents from the same manufacturer.

For complete blood count (white blood count, eosinophil count, eosinophil percentage, neutrophil count, red blood count, and platelet), the blood samples were collected in tubes with EDTA and analyzed on CELL DYN 4000 Abbott analyzer (Abbott Diagnositics, Santa Clara, California, USA), which was calibrated daily.

### National institutes of health stroke scale

NIHSS is of predictive value for clinical outcome and therapy in AIS [10]. NIHSS may be performed rapidly, have demonstrated utility, and may be administered by a broad spectrum of healthcare providers (Table 6) [9–11]. The NIHSS was administered by physicians certified in its administration on admission and discharge. NIHSS score was calculated from the documented neurological examination of the treating neurologist in the medical records, and by a neurologist.

**Table 6:** National institutes of health stroke scale

Tested item	Title	Responses and scores
1A	Level of consciousness	0-Alert 1-Drowsy 2-Obtunded 3-Coma/unresponsive
1B	Orientation questions	0-Answers both correctly 1-Answers 1 correctly 2-Answers neither correctly
1C	Responses to commands	0-Performs both tasks correctly 1-Performs 1 task correctly 2-Performs neither
2	Gaze	0-Normal horizontal movements 1-Partial gaze palsy 2-Complete gaze palsy
3	Visual	0-No visual field defect 1-Partial hemianopia 2-Complete hemianopia 3-Bilateral hemianopia
4	Facial movement	0-Normal 1-Minor facial weakness 2-Partial facial weakness 3-Complete unilateral palsy
5	Motor function(arm) a. Left b. Right	0-No drift 1-Drift before 5 seconds 2-Falls before 10 seconds 3-No effort against gravity 4-No movement
6	Motor function(leg) a. Left b. Right	0-No drift 1-Drift before 5 seconds 2-Falls before 5 seconds 3-No effort against gravity 4-No movement
7	Limb ataxia	0-No ataxia 1-Ataxia in 1 limb 2-Ataxia in 2 limb
8	Sensory	0-No sensory loss 1-Mild sensory loss 2-Severe sensory loss
9	Language	0-Normal 1-Mild aphasia 2-Severe aphasia 3-Mute or global aphasia
10	Articulation	0-Normal 1-Mild dysarthria 2-Severe dysarthria
11	Extinction or inattention	0-Absent 1-Mild(loss 1 sensory modality lost) 2-Severe(loss 2 modalities lost)

### Statistical analysis

Statistical analyses were carried out using SPSS 19.0 (SPSS Inc., Chicago, Illinois, USA). Baseline data of patients were presented as the means±standard deviation and compared using student’s *t* test for continuous variables and the chi-squared test was used for non-continuous variables. Multi-factorial analysis using the backward stepwise logistic regression was used for in-hospital death. Correlation was assessed by spearson’s correlation coefficient or Wilcoxon test. Multiple stepwise linear regression analysis was used for predictors of NIHSS. Receiver-operating characteristics (ROC) analyses were used to detect the cut-off values of eosinophils and NIHSS in the prediction of non-death; sensitivity and specificity at that point were determined. *P* value <0.05 was considered statistically significant.

## CONCLUSIONS

Our study revealed a relationship between eosinophil and NIHSS score in the patients with AIS. Eosinophils may play a certain role in the process of the occurrence of AIS.

## References

[1] Mozaffarian D, Benjamin EJ, Go AS, Arnett DK, Blaha MJ, Cushman M, Das SR, de Ferranti S, Després JP, Fullerton HJ, Howard VJ, Huffman MD, Isasi CR, Writing Group Members, and American Heart Association Statistics Committee, and Stroke Statistics Subcommittee (2016). Heart Disease and Stroke Statistics-2016 Update: A Report From the American Heart Association. Circulation.

[2] Bentzon JF, Otsuka F, Virmani R, Falk E (2014). Mechanisms of plaque formation and rupture. Circ Res.

[3] Hernández-Jiménez E, Gutierrez-Fernández M, Cubillos-Zapata C, Otero-Ortega L, Rodríguez-Frutos B, Toledano V, Martínez-Sánchez P, Fuentes B, Varela-Serrano A, Avendaño-Ortiz J, Blázquez A, Mangas-Guijarro MA, Díez-Tejedor E, López-Collazo E (2017). Circulating Monocytes Exhibit an Endotoxin Tolerance Status after Acute Ischemic Stroke: Mitochondrial DNA as a Putative Explanation for Poststroke Infections. J Immunol.

[4] Pagram H, Bivard A, Lincz LF, Levi C (2016). Peripheral Immune Cell Counts and Advanced Imaging as Biomarkers of Stroke Outcome. Cerebrovasc Dis Extra.

[5] Wang JG, Mahmud SA, Thompson JA, Geng JG, Key NS, Slungaard A (2006). The principal eosinophil peroxidase product, HOSCN, is a uniquely potent phagocyte oxidant inducer of endothelial cell tissue factor activity: a potential mechanism for thrombosis in eosinophilic inflammatory states. Blood.

[6] Samoszuk M, Corwin M, Hazen SL (2003). Effects of human mast cell tryptase and eosinophil granule proteins on the kinetics of blood clotting. Am J Hematol.

[7] Sarazin M, Caumes E, Cohen A, Amarenco P (2004). Multiple microembolic borderzone brain infarctions and endomyocardial fibrosis in idiopathic hypereosinophilic syndrome and in Schistosoma mansoni infestation. J Neurol Neurosurg Psychiatry.

[8] Moore PM, Harley JB, Fauci AS (1985). Neurologic dysfunction in the idiopathic hypereosinophilic syndrome. Ann Intern Med.

[9] Yao S, Zhu Y, Chen L (2013). Advances in targeting cell surface signalling molecules for immune modulation. Nat Rev Drug Discov.

[10] Brott T, Adams HP, Olinger CP, Marler JR, Barsan WG, Biller J, Spilker J, Holleran R, Eberle R, Hertzberg V (1989). Measurements of acute cerebral infarction: a clinical examination scale. Stroke.

[11] Agis D, Goggins MB, Oishi K, Oishi K, Davis C, Wright A, Kim EH, Sebastian R, Tippett DC, Faria A, Hillis AE (2016). Picturing the Size and Site of Stroke With an Expanded National Institutes of Health Stroke Scale. Stroke.

[12] Weller PF, Bubley GJ (1994). The idiopathic hypereosinophilic syndrome. Blood.

[13] Greco A, Rizzo MI, De Virgilio A, Gallo A, Fusconi M, Ruoppolo G, Altissimi G, De Vincentiis M (2015). Churg-Strauss syndrome. Autoimmun Rev.

[14] Slungaard A, Vercellotti GM, Tran T, Gleich GJ, Key NS (1993). Eosinophil cationic granule proteins impair thrombomodulin function. A potential mechanism for thromboembolism in hypereosinophilic heart disease. J Clin Invest.

[15] Slungaard A, Vercellotti GM, Walker G, Nelson RD, Jacob HS (1990). Tumor necrosis factor alpha/cachectin stimulates eosinophil oxidant production and toxicity towards human endothelium. J Exp Med.

[16] Sakai T, Inoue S, Matsuyama TA, Takei M, Ota H, Katagiri T, Koboyashi Y (2009). Eosinophils may be involved in thrombus growth in acute coronary syndrome. Int Heart J.

[17] Jiang P, Wang DZ, Ren YL, Cai JP, Chen BX (2015). Significance of eosinophil accumulation in the thrombus and decrease in peripheral blood in patients with acute coronary syndrome. Coron Artery Dis.

[18] Shafqat S, Kvedar JC, Guanci MM, Chang Y, Schwamm LH (1999). Role for telemedicine in acute stroke. Feasibility and reliability of remote administration of the NIH stroke scale. Stroke.

[19] Adams HP, Davis PH, Leira EC, Chang KC, Bendixen BH, Clarke WR, Woolson RF, Hansen MD (1999). Baseline NIH Stroke Scale score strongly predicts outcome after stroke: A report of the Trial of Org 10172 in Acute Stroke Treatment (TOAST). Neurology.

[20] Frankel MR, Morgenstern LB, Kwiatkowski T, Lu M, Tilley BC, Broderick JP, Libman R, Levine SR, Brott T (2000). Predicting prognosis after stroke: a placebo group analysis from the National Institute of Neurological Disorders and Stroke rt-PA Stroke Trial. Neurology.

[21] Swaminathan GJ, Weaver AJ, Loegering DA, Checkel JL, Leonidas DD, Gleich GJ, Acharya KR (2001). Crystal structure of the eosinophil major basic protein at 1.8 A. An atypical lectin with a paradigm shift in specificity. J Biol Chem.

[22] Fredens K, Dahl R, Venge P (1991). In vitro studies of the interaction between heparin and eosinophil cationic protein. Allergy.

[23] Hällgren R, Venge P, Cullhed I, Olsson I (1979). Blood eosinophils and eosinophil cationic protein after acute myocardial infarction or corticosteroid administration. Br J Haematol.

[24] Weiss SJ, Test ST, Eckmann CM, Roos D, Regiani S (1986). Brominating oxidants generated by human eosinophils. Science.

[25] Wu W, Chen Y, Hazen SL (1999). Eosinophil peroxidase nitrates protein tyrosyl residues. Implications for oxidative damage by nitrating intermediates in eosinophilic inflammatory disorders. J Biol Chem.

[26] Egoz N, Shmilovitz M, Kretzer B, Lucian M, Porat V, Raz R (1991). An outbreak of Shigella sonnei infection due to contamination of a municipal water supply in northern Israel. J Infect.

[27] Arlandson M, Decker T, Roongta VA, Bonilla L, Mayo KH, MacPherson JC, Hazen SL, Slungaard A (2001). Eosinophil peroxidase oxidation of thiocyanate. Characterization of major reaction products and a potential sulfhydryl-targeted cytotoxicity system. J Biol Chem.

[28] Grisham MB, Ryan EM (1990). Cytotoxic properties of salivary oxidants. Am J Physiol.

[29] Cadroy Y, Dupouy D, Boneu B, Plaisancié H (2000). Polymorphonuclear leukocytes modulate tissue factor production by mononuclear cells: role of reactive oxygen species. J Immunol.

[30] Sugiyama S, Kugiyama K, Aikawa M, Nakamura S, Ogawa H, Libby P (2004). Hypochlorous acid, a macrophage product, induces endothelial apoptosis and tissue factor expression: involvement of myeloperoxidase-mediated oxidant in plaque erosion and thrombogenesis. Arterioscler Thromb Vasc Biol.

